# Serum Metabolite Biomarkers Predictive of Response to PD-1 Blockade Therapy in Non-Small Cell Lung Cancer

**DOI:** 10.3389/fmolb.2021.678753

**Published:** 2021-05-21

**Authors:** Xiaoqun Nie, Liliang Xia, Fang Gao, Lixia Liu, Yi Yang, Yingying Chen, Huangqi Duan, Yaxian Yao, Zhiwei Chen, Shun Lu, Ying Wang, Chen Yang

**Affiliations:** ^1^ CAS-Key Laboratory of Synthetic Biology, CAS Center for Excellence in Molecular Plant Sciences, Chinese Academy of Sciences, Shanghai, China; ^2^ Department of Shanghai Lung Cancer, Shanghai Chest Hospital, Shanghai Jiao Tong University, Shanghai, China; ^3^ Department of Immunology and Microbiology, Shanghai Institute of Immunology, Shanghai Jiao Tong University School of Medicine, Shanghai, China

**Keywords:** immune checkpoint inhibitors, non-small cell lung cancer, serum metabolomics, metabolite biomarker, non-targeted metabolomics

## Abstract

**Background:** Despite remarkable success of immunotherapies with checkpoint blockade antibodies targeting programmed cell death protein 1 (PD-1), the majority of patients with non-small-cell lung cancer (NSCLC) have yet to receive durable benefits. We used the metabolomic profiling of early on-treatment serum to explore predictors of clinical outcomes of anti-PD-1 treatment in patients with advanced NSCLC.

**Methods:** We recruited 74 Chinese patients who had stage IIIB/IV NSCLC-proven tumor progression and were treated with PD-1 inhibitor. The study was comprised of a discovery cohort of patients treated with nivolumab and two validation cohorts of patients receiving tislelizumab or nivolumab. Serum samples were collected 2–3 weeks after the first infusion of PD-1 inhibitor. Metabolomic profiling of serum was performed using ultrahigh performance lipid chromatograph-mass spectrometry. The serum metabolite biomarkers were identified using an integral workflow of nontargeted metabolomic data analysis.

**Results:** A serum metabolite panel consisting of hypoxanthine and histidine was identified and validated as a predictor of response to PD-1 blockade treatment in patients with advanced NSCLC. High levels of both hypoxanthine and histidine in early on-treatment serum were associated with improved progression-free survival [hazard ratio (HR) = 0.078, 95% confidence interval (CI), 0.027–0.221, *p <* 0.001] and overall survival (HR = 0.124, 95% CI, 0.039–0.397, *p <* 0.001) in the discovery cohort. The serum metabolite panel showed a high sensitivity and specificity in distinguishing responders and non-responders in the validation cohorts 1 and 2, with an area under the receiver-operating characteristic curve of 0.933 and 1.000, respectively. High levels of serum hypoxanthine and histidine were correlated with improved progression-free survival in the validation cohort 1 (HR = 0.137, 95% CI, 0.040–0.467, *p =* 0.001) and in the validation cohort 2 (HR = 0.084, 95% CI, 0.009–0.762, *p =* 0.028).

**Conclusion:** Our results revealed that hypoxanthine and histidine in early on-treatment serum are predictive biomarkers of response to PD-1 blockade therapy in patients with advanced NSCLC. The serum biomarker panel would enable early identification of NSCLC patients who may benefit from PD-1 blockade therapy.

## Introduction

Non-small cell lung cancer (NSCLC) is the leading cause of cancer-related mortality worldwide and generally has a poor prognosis (Bray et al., 2018). During the past several years, major advances have been made in cancer treatment through the use of immune checkpoint inhibitors (ICIs) (Ribas and Wolchok, 2018). ICIs targeting programmed cell death protein 1 (PD-1) or its ligand PD-L1 have demonstrated improved clinical efficacy in both second-line and first-line treatment of advanced NSCLC when compared to conventional chemotherapy (Sui et al., 2018). At present, two anti-PD-1 antibodies nivolumab and pembrolizumab as well as several anti-PD-L1 antibodies have been approved by the United States. Food and Drug Administration (FDA) for treatment of multiple cancer types including NSCLC (Ribas and Wolchok, 2018). Recently, an anti-PD-1 antibody tislelizumab has been approved in China for treatment of NSCLC and other cancers (Liu and Wu, 2020). These PD-1/PD-L1 inhibitors block the binding of PD-1 to its PD-L1 ligand and restore the capacity of cytotoxic T cells to recognize and kill cancer cells. Current PD-1/PD-L1 blockade therapies have shown durable disease control and improved survival in patients with advanced NSCLC (Rangachari and Costa, 2019). However, only subsets of patients are benefiting from the anti-PD-1/PD-L1 therapies. For example, only 10–30% of patients with NSCLC have objective tumor responses to treatment with nivolumab (Borghaei et al., 2015; Topalian et al., 2019). The mechanistic basis for the variation in response patterns remains poorly explained. In addition, some patients experience severe autoimmune adverse events (Friedman et al., 2016; Spain et al., 2016). Given the distinct response patterns, combined with potentially severe toxicity and high costs, there is an urgent need to identify biomarkers that can predict which patients are likely to benefit from PD-1/PD-L1 blockade therapies.

So far, PD-L1 expression, which is assayed by immunohistochemistry (IHC) staining on tumor specimens, is the most commonly used biomarker for selecting patients treated with anti-PD-1/PD-L1 antibodies (Topalian et al., 2015). However, PD-L1 expression was not consistently associated with tumor responses and patient survival. For example, only 44.8% of PD-L1-positive NSCLCs are responsive to pembrolizumab in a first-line treatment (Garon et al., 2015). A proportion of PD-L1-negative patients with NSCLC or other cancers also benefits from anti-PD-1 therapy (Robert et al., 2015). Several other biomarkers, which include tumor mutational load, mismatch-repair deficiency, neoantigens, density of tumor-infiltrating lymphocytes, and the diversity of gut microbiome, have been reported to correlate with the clinical outcomes (Le et al., 2015; Rizvi et al., 2015; Berghoff et al., 2016; McGranahan et al., 2016; Jin et al., 2019). However, these proposed biomarkers are not perfectly predictive. Moreover, most of them are based on tumor assays, which require invasive sampling, and are not practical for monitoring tumor response during treatment. Recently, circulating blood biomarkers for prediction of immunotherapeutic responses have attracted increasing attention because they can be minimally invasively obtained from patients and trended over time (Li, Bullock, et al., 2019).

Tumor-infiltrating immune cells typically experience metabolic stress as a result of the dysregulated metabolic activity of tumor cells, which can result in immunosuppression and tumor immune evasion (Herbel et al., 2016). Cumulative evidence indicates that combination of ICIs with interventions targeting the metabolic circuits that impede antitumour immunity may be a promising strategy to improve clinical efficacy (Li, Wenes, et al., 2019). Metabolic biomarkers of immunotherapeutic responses can not only guide the therapeutic decisions but also lead to identification of novel metabolic targets for combination therapies. Advances in mass spectrometry (MS)-based metabolomics have allowed the discovery of new biomarkers for cancer diagnosis and customized treatment (Crutchfield et al., 2016). However, up to now, only a few metabolomics studies have been performed to investigate the changes in serum metabolites after anti-PD-1 treatment (Li H, et al., 2019), the gut microbiota-derived metabolites in responsive patients (Frankel et al., 2017), and the correlation between plasma metabolites and T cell markers (Hatae et al., 2020). Metabolic biomarkers that can reliably predict outcomes of anti-PD-1/PD-L1 treatments remain to be uncovered.

In this study, by comprehensively profiling metabolites in early on-treatment serum from a discovery cohort, we identified a metabolite panel consisting of hypoxanthine and histidine as a predictor of NSCLC response to PD-1 blockade, which was then validated in independent patient cohorts. High levels of the serum metabolite biomarkers were found to correlate with improved survival of patients with NSCLC receiving PD-1 blockade therapy.

## Materials and Methods

### Patients

Patients of this study were recruited from Shanghai Chest Hospital affiliated to Shanghai Jiao Tong University (Shanghai, China). All the participants had histologically proven stage IIIB/IV NSCLC (Table 1). Serum samples from a patient cohort treated with nivolumab were used as a discovery set to identify potential serum biomarkers of response to PD-1 blockade therapy. The potential metabolite biomarkers were validated in a patient cohort receiving tislelizumab and another cohort treated with nivolumab. The patients treated with nivolumab had squamous or non-squamous cell carcinoma and had received one to two prior systemic therapies and proved progression before PD-1 blockade therapy. The patients receiving tislelizumab all had non-squamous cell carcinoma, and tislelizumab was used as first-line therapy in combination with chemotherapy. All the participants in this study were followed up until disease progression or death. Patients received nivolumab (240 mg) every 2 weeks, and tislelizumab (200 mg) was administered every 3 weeks. Peripheral blood samples were collected after administration of nivolumab or tislelizumab. Disease severity was measured by computed tomography or magnetic resonance imaging and evaluated for therapeutic response using Response Evaluation Criteria in Solid Tumors 1.1 (RECIST 1.1). Clinical response to the treatment with nivolumab or tislelizumab was evaluated every 8 weeks and was confirmed by a subsequent assessment no less than 4 weeks thereafter. Electronic medical charts were reviewed independently by two investigators to assign clinical response groups. Responders were defined by freedom from disease, stable disease, or decreased tumor volume for more than 6 months, and non-responders were defined by tumor growth or a clinical benefit lasting 6 months or less (Hodi et al., 2018). Patients gave their written informed consent to participate in the research, which had received approval from the Ethics Committee of Shanghai Chest Hospital. All the procedures were conducted in accordance with the Declaration of Helsinki.

**TABLE 1:** Clinical characteristics of discovery and validation sets and efficacy of anti-PD-1 therapy.

Characteristics^a^	Discovery set (*n* = 43)	Validation set 1 (*n* = 21)	Validation set 2 (*n* = 10)
Age, year	63 (41–74)	60.4 (54–72)	64.5 (46–78)
Sex			
Male	33 (77%)	14 (67%)	9 (90%)
Female	10 (23%)	7 (33%)	1 (10%)
Smoking status			
Smoker	31 (72%)	11 (52%)	8 (80%)
Non-smoker	12 (28%)	10 (48%)	2 (20%)
Histology			
Squamous	17 (40%)	0	7 (70%)
Non-squamous	26 (60%)	21 (100%)	3 (30%)
Disease stage			
III	6 (14%)	1 (5%)	1 (10%)
IV	37 (86%)	20 (95%)	9 (90%)
Metastasis			
Yes	37 (86%)	20 (95%)	9 (90%)
No	6 (14%)	1 (5%)	1 (10%)
Previous chemotherapy treatment			
Cisplatin based	28 (62%)	-	5 (36%)
Carboplatin based	12 (27%)	-	6 (43%)
Others	3 (7%)	-	3 (21%)
No previous treatment	0	-	0
Unknown	2 (4%)	-	0
Radiotherapy			
Yes	15 (35%)	4 (19%)	8 (80%)
No	27 (63%)	9 (43%)	2 (20%)
Unknown	1 (2%)	8 (38%)	0
Clinical benefit to PD-1 blockade			
Durable clinical benefit	23 (53%)	13 (62%)	4 (40%)
No clinical benefit	20 (47%)	8 (38%)	6 (60%)
RECIST response to PD-1 blockade			
Complete response	0	0	0
Stable disease	18 (42%)	6 (29%)	6 (60%)
Partial response	8 (19%)	13 (62%)	0
Progression disease	17 (39%)	2 (9%)	4 (40%)
Progression-free survival since PD-1 blockade, days	152 (24–645)	369 (42–770)	87 (27–429)
Overall survival since PD-1 blockade, days	573 (33–648)	596 (86–769)	366 (144–429)

a Data are expressed as number (%) or median (range).

### Sample Preparation

Serum was collected after centrifugation of peripheral blood at 1500 g for 10 min and immediately stored at −80°C. The samples were thawed on ice. Then 100 μl of samples were mixed with 50 μl of internal standard (6 μg/ml 2-chloro-L-phenylalanine in water) and 350 μl of methanol. After vortex for 1 min, the samples were centrifuged at 14,000 g for 15 min, and the supernatant was used for LC-MS analysis. Quality control (QC) samples were prepared by mixing aliquots of serum samples from a subset of the cohort and using the same procedure as the samples studied.

### Liquid Chromatography-Mass Spectrometry Analysis

Metabolites were profiled using ultrahigh performance lipid chromatography-mass spectrometry (UHPLC-MS). Samples were injected onto a UHPLC system (Acquity, Waters) coupled to a Q Exactive hybrid quadrupole-orbitrap mass spectrometer (Thermo Fisher). The sample injection order was randomized, and QC and blank samples (80% methanol in water) were regularly injected throughout the run. The injection volume was 10 μl. Metabolites were separated with a Luna NH2 column (50 mm × 2 mm, 5 μm particle size, Phenomenex) (Yuan et al., 2012). The column was maintained at 15°C with a solvent flow rate of 0.3 ml/min. Solvent A was 20 mM ammonium acetate adjusted to pH 9.0 with ammonium hydroxide, and solvent B was acetonitrile. The gradient of B was as follows: 0 min, 85%; 3 min, 30%; 12 min, 2%; 15 min, 2%; 16 min, 85%; 23 min, 85% B. The mass spectrometer was run in both electrospray ionization positive (ESI^+^) and negative (ESI^−^) modes. The key parameters were as follows: ionization voltage, +3.8 kV/−3.8 kV; sheath gas pressure, 35 arbitrary units; capillary temperature, 320°C. The mass spectrometer was run in full scan mode at an *m/z* 70–1000 scan range and 70,000 resolution. MS/MS spectra were acquired with 15–35-eV collision energy.

### Data Processing

Data processing was performed using an integral workflow of nontargeted metabolomic data analysis (Dunn et al., 2011). Briefly, data were processed by R package XCMS, followed by quality checks and signal drift correction to generating a data matrix that consisted of retention time, *m/z* value, and peak intensity. The peak area of each metabolite was normalized to sum of areas of all metabolites present in the sample, and then unit-variance scaled before further statistical analysis (Gorrochategui et al., 2016). The accurate mass and acquired MS/MS spectra were used for metabolite identification by matching with in-house spectral libraries and online databases (mzCloud, MoNA, and HMDB) (Kind et al., 2018). Quantitation of serum metabolites was performed by using a targeted analysis and external calibration curves as reported previously (Roberts et al., 2012).

### Statistical Analysis

Multivariate statistical analysis of metabolomic data was performed using the SIMCA software (Umetrics). Unsupervised principal component analysis was conducted to visualize grouping trends and the clustering of QC samples. A supervised model of orthogonal partial least-squares-discriminant analysis (OPLS-DA) was applied to identify the metabolites contributing to class separation according to corresponding variable importance in the projection (VIP). The OPLS-DA parameters, R2Y and Q2, were used for evaluating the goodness of the model fit. The risk of overfitting of the OPLS-DA model was evaluated by performing 200 permutation tests.

Univariate statistical analysis of marker metabolites was performed using the Multi Experimental Viewer software (http://www.tm4.org). A nonparametric Wilcoxon-Mann-Whitney test was conducted, and a *p* value < 0.05 was considered a priori to be statistically significant. The metabolites with false discovery rate (FDR) values less than 0.05 and VIP values greater than 1.0 were defined as putative marker metabolites. The biomarker model was built by binary logistic regression using forward stepwise method. To evaluate the classification performance, receiver operating characteristic (ROC) analysis was conducted and the area under the ROC curve (AUC) was computed by using the MedCalc software (https://www.medcalc.org/).

The Kaplan-Meier method was used to estimate progression-free and overall survival, with the differences between the groups calculated with the log-rank test. Hazard ratios (HRs) from univariate Cox regression were used to determine the association between marker metabolites and survival. Multivariate Cox regression was conducted to adjust for patient characteristics by using the SPSS software (SPSS Inc.).

## Results

### Study Population

The characteristics of the study cohorts are summarized in Table 1. To identify potential serum biomarkers of clinical response to PD-1 blockade, we collected serum samples from a discovery cohort of 43 patients with advanced NSCLC treated with the anti-PD-1 antibody nivolumab. The potential biomarkers were confirmed in two independent validation sample sets. A validation cohort was comprised of 21 patients with advanced NSCLC treated with another PD-1 inhibitor, tislelizumab. Another cohort for biomarker validation includes 10 patients with NSCLC treated with nivolumab. Nivolumab-treated patients had squamous or non-squamous cell carcinoma, whereas all the patients receiving tislelizumab had non-squamous cell carcinoma. The patients were treated with nivolumab as the second-line or third-line therapy, whereas tislelizumab was used in the first-line combination therapy of the patients. We defined responsive and non-responsive patients based on the following criteria (Hodi et al., 2018): patients with durable clinical benefit (defined as no progression event or death within the first 6 months of PD-1 blockade) were classified as responders; patients with no durable clinical benefit (progression event or death within the first 6 months of PD-1 blockade) were classified as non-responders. No significant difference was observed in age, sex, disease history, disease stage, smoking history, and prior treatments between responders and non-responders in nivolumab- or tislelizumab-treated patients (Supplementary Table S1).

### Identification of Potential Metabolite Biomarkers of Response to PD-1 Blockade

**FIGURE 1:**
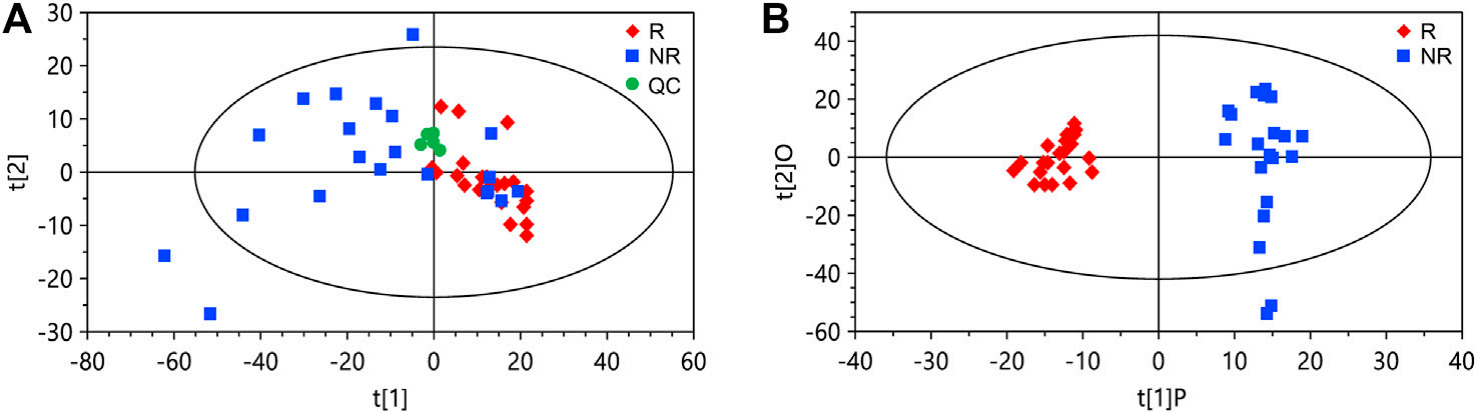
Principal component analysis (PCA) and orthogonal partial least squares discriminant analysis (OPLS-DA) of the data from the discovery set. **(A)** PCA score plot. **(B)** OPLS-DA score plot. The cumulative *R* ^2^ *Y* and *Q* ^2^ *Y* of the OPLS-DA model are 0.92 and 0.67, respectively. R, responders; NR, non-responders; QC, quality control.

We collected serum samples from the discovery cohort 2 weeks after the first infusion of nivolumab. Among the patients in the discovery cohort (*n =* 43), 21 being evaluated as partial response or stable disease were classified as responders, and 22 with disease progression were classified as non-responders. By using ultrahigh performance lipid chromatography-mass spectrometry (UHPLC-MS), a total of 1,566 metabolite peaks were detected, including 803 in the negative and 763 in the positive ionization modes. The quality control samples are clustered in the score plot of principal component analysis (PCA), indicating the good reproducibility of the metabolomics analysis (Figure 1A). The PCA of the discovery set also demonstrated a tendency of difference in metabolomic profiles between responders and non-responders. For screening of potential marker metabolites, orthogonal partial least squares discriminant analysis (OPLS-DA) was applied. The OPLS-DA score plot of the discovery set revealed a clear separation between responders and non-responders without overfitting (Figure 1B). The validity of the OPLS-DA model was confirmed using permutation tests (Supplementary Figure S1). A subsequent univariate analysis was performed, resulting in identification of 185 metabolite peaks with a variable importance in the project (VIP) > 1.0, *p <* 0.05, and a false discovery rate (FDR) < 0.05 as important variables contributing to class separation.

**FIGURE 2:**
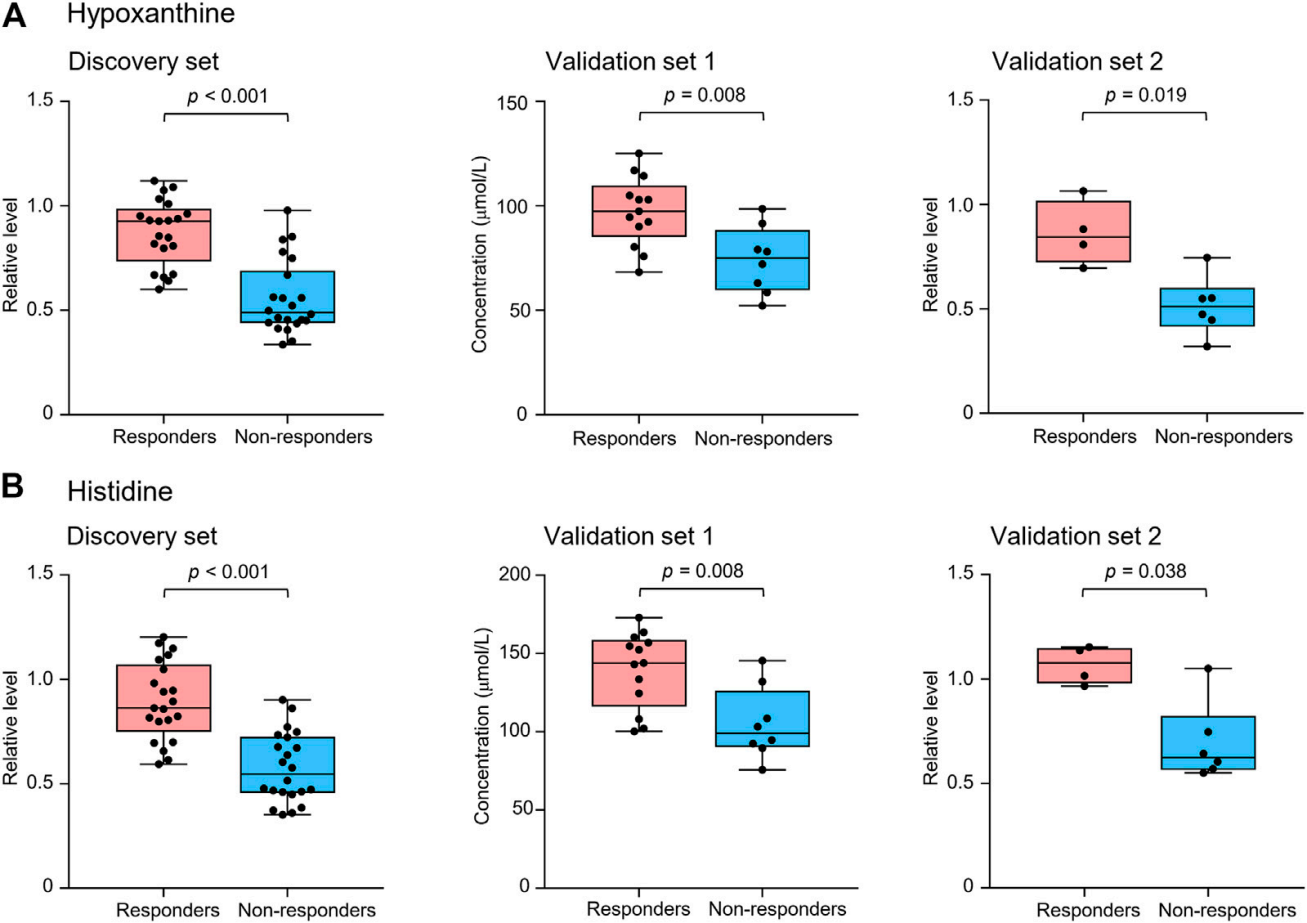
Serum levels of potential marker metabolites hypoxanthine **(A)** and histidine **(B)** at early on-treatment in responders and non-responders of the discovery set and validation sets 1 and 2. The box plots depict the minimum and maximum values (whiskers), the upper and lower quartiles, and the median. Groups were compared by Wilcoxon-Mann-Whitney test with Benjamini-Hochberg-based adjustment for multiple comparisons.

**FIGURE 3:**
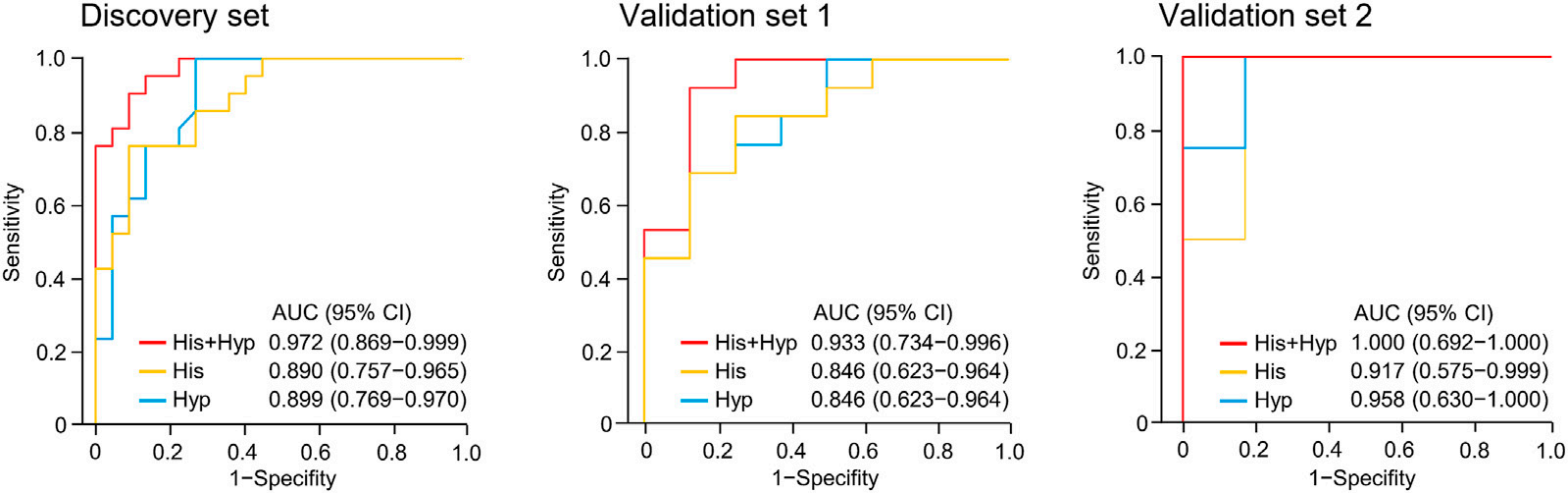
Receiver operating characteristic (ROC) analysis of hypoxanthine and/or histidine in the discovery set and validation sets 1 and 2. Hyp, hypoxanthine; His, histidine; AUC, area under the curve.

Metabolite identification was performed by matching accurate mass and tandem MS/MS spectra with in-house spectral libraries and online databases and by confirmation with authentic standards. Thus, six candidates of marker metabolites were obtained, including cystine, threonine, histidine, 3-oxotetradecanoic acid, 1,7-dimethyluric acid, and hypoxanthine (Supplementary Table S2). Binary logistic regression was performed to construct the best model using the six metabolites (Supplementary Table S3). Therefore, the combination of hypoxanthine and histidine was identified as the best biomarker panel to distinguish responders and non-responders to nivolumab treatment. Responders had significantly higher levels of both marker metabolites in early on-treatment serum than non-responders (*p <* 0.001 and *p <* 0.001, respectively) (Figure 2). By contrast, the PD-L1 expression in pretreatment tumors was not significantly different (*p =* 0.116) between responders and non-responders in the discovery cohort (Supplementary Table S1). The receiver operating characteristic (ROC) analysis showed that the metabolite panel performs better than each metabolite in discrimination of responders and non-responders (Figure 3). The area under the curve (AUC) for the metabolite panel was 0.972 [95% confidence interval (CI), 0.869–0.999], with sensitivity of 95% and specificity of 86%.

### High Marker Metabolite Levels Correlate With Improved Patient Survival

**FIGURE 4:**
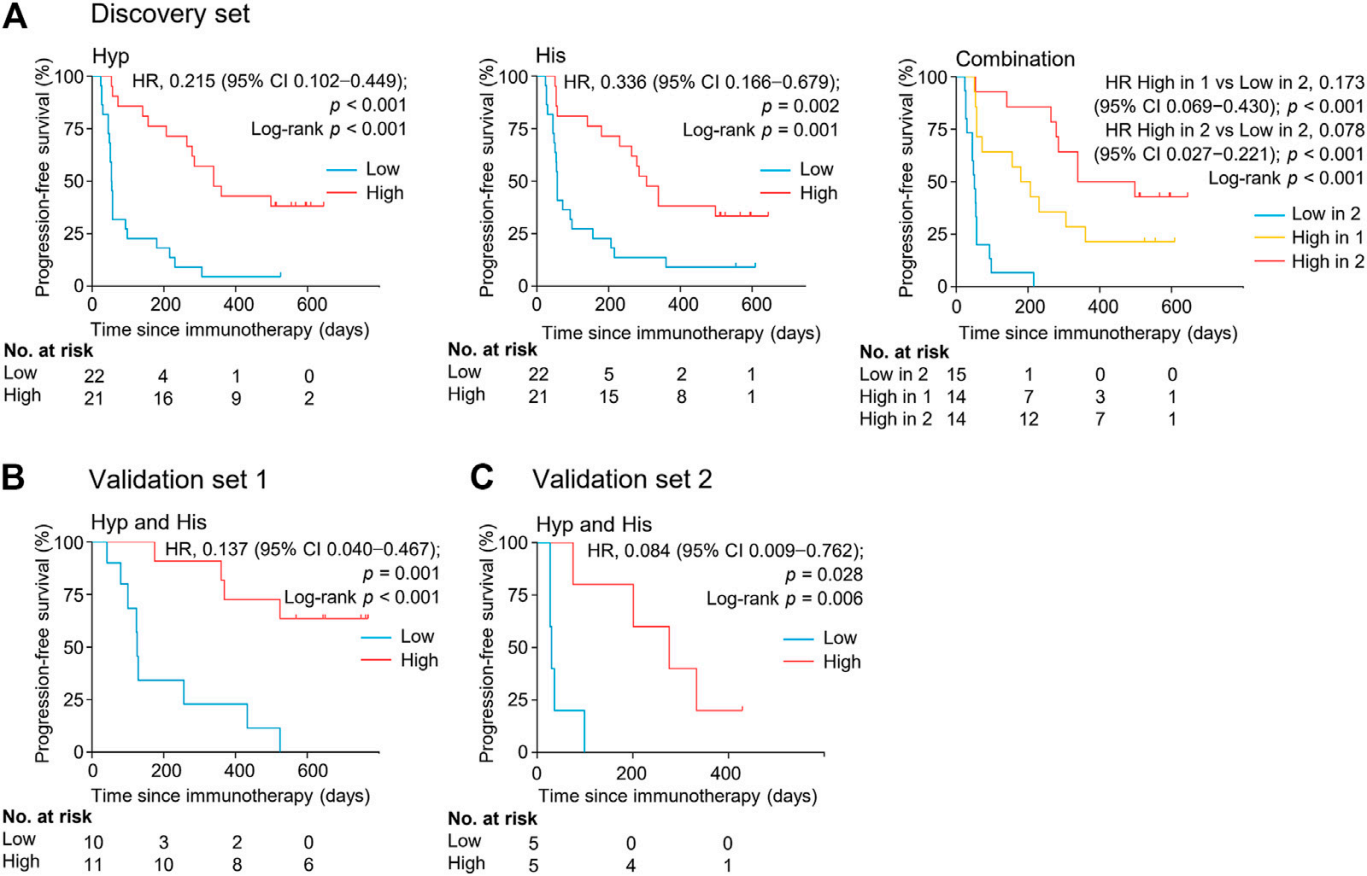
Serum levels of metabolite biomarkers at early on-treatment associate with progression-free survival in the discovery set **(A)**, validation set 1 **(B)**, and validation set 2 **(C)**. Kaplan-Meier analysis for progression-free survival in NSCLC patients by serum levels of hypoxanthine and/or histidine. His, histidine; Hyp, hypoxanthine; HR, hazard ratio.

The association between the marker metabolites and the clinical outcome of nivolumab treatment was examined. Metabolite levels were dichotomized into high and low categorical variables based on the median value in the analyzed samples. We found that the serum levels of hypoxanthine and histidine at early on-treatment were significantly associated with progression-free survival (PFS) [hazard ratio (HR) = 0.215, 95% CI, 0.102–0.449, *p <* 0.001; HR = 0.336, 95% CI, 0.166–0.679, *p =* 0.002, respectively] (Figure 4, Supplementary Table S4). The combination of both metabolites and the association with PFS demonstrated an additive effect (HR = 0.078, 95% CI, 0.027–0.221, *p* < 0.001) (Figure 4, Supplementary Table S4). The median PFS of patients with high levels of one or both metabolites were 180 days (95% CI, 57–360 days) and 339 days (95% CI, 264–498 days), respectively, whereas patients with low levels of both metabolites had a median PFS of 51 days (95% CI, 27–57 days) (*p <* 0.001) (Figure 4). The combination of both metabolites remained an independent factor for PFS in the multivariate analysis (Supplementary Table S4).

**FIGURE 5:**
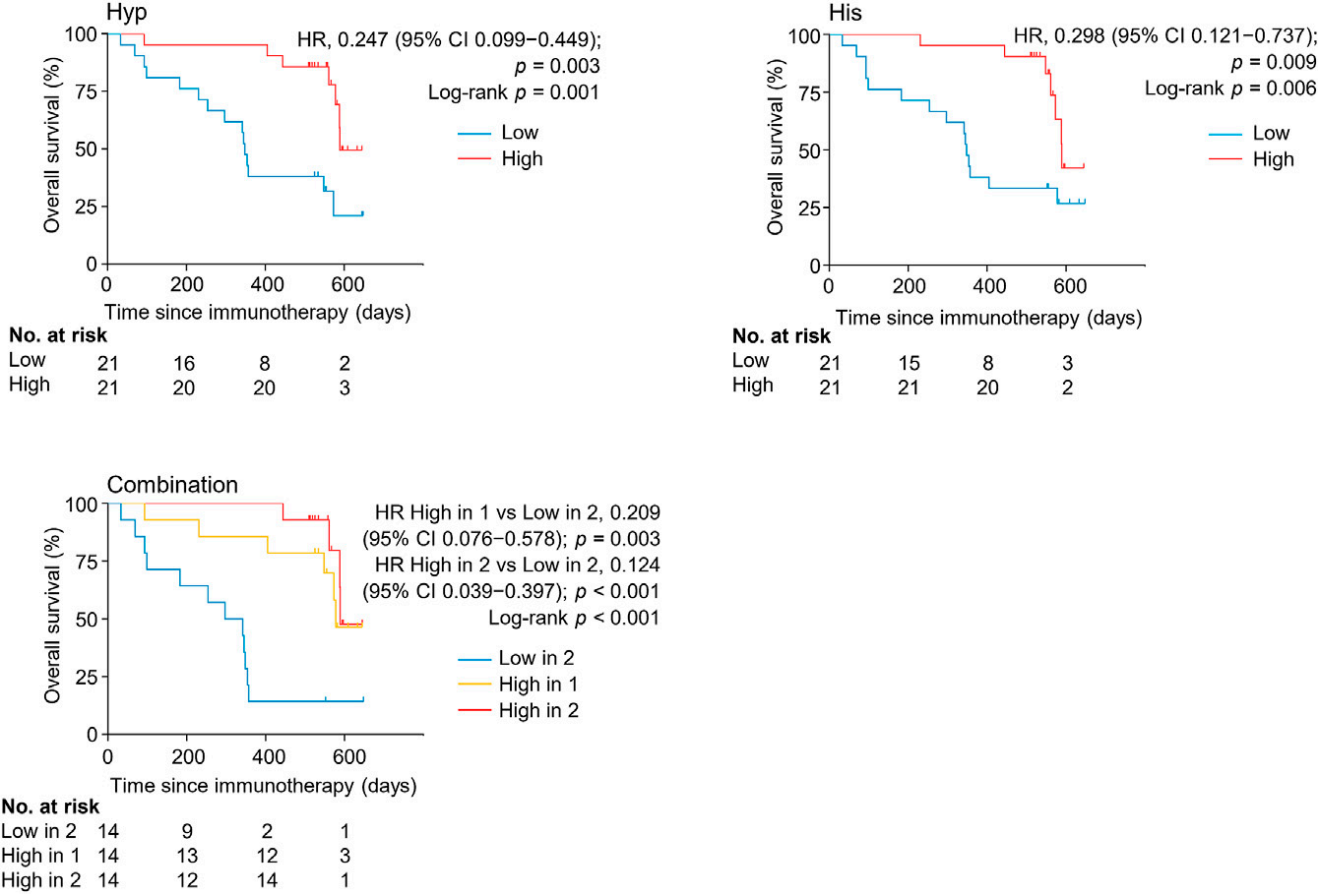
Kaplan-Meier estimates of overall survival by serum levels of hypoxanthine and/or histidine in the discovery set. His, histidine; Hyp, hypoxanthine; HR, hazard ratio.

The levels of serum hypoxanthine and histidine were also significantly and independently correlated with overall survival (OS) (HR = 0.124, 95% CI, 0.039–0.397, *p <* 0.001) (Figure 5, Supplementary Table S5). Patients with high levels of both metabolites had a longer overall survival (median OS of 589 days, 95% CI, 56–589 days) than did patients with low levels of both metabolites (median OS of 297 days, 95% CI 93–354 days) (*p <* 0.001). Thus, hypoxanthine and histidine in early on-treatment serum were identified as the potential biomarkers predictive of clinical outcomes in patients with NSCLC receiving PD-1 blockade therapy.

### Validation of Serum Metabolite Biomarkers Predictive of Response to PD-1 Blockade

**FIGURE 6:**
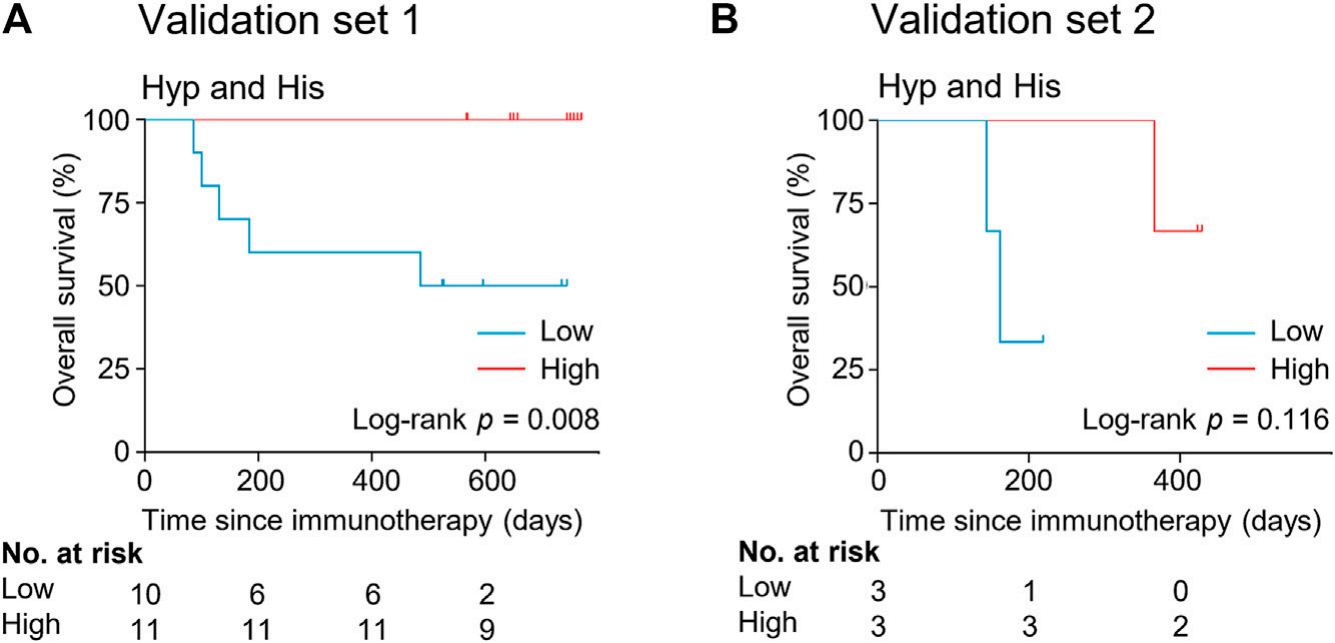
Kaplan-Meier estimates of overall survival by serum levels of hypoxanthine and histidine in the validation set 1 **(A)** and validation set 2 **(B)**. His, histidine; Hyp, hypoxanthine.

The potential metabolite biomarkers were evaluated in two independent validation sample sets. Serum samples were collected from a validation cohort 3 weeks after the first infusion of tislelizumab. This cohort consisted of 13 responders and eight non-responders (validation set 1) (Table 1). We determined the absolution concentrations of hypoxanthine and histidine in serum (Figure 2), and the measurements were highly reproducible. The logistic regression model of the metabolite panel for predicting response to PD-1 blockade was constructed as follows: logit (*p =* Responder) = 0.114 × (Hyp) + 0.079 × (His) − 19.548. In this equation, (*p =* Responder) is the predicted probability of NSCLC patients benefiting from PD-1 blockade therapy, (Hyp) and (His) are the serum concentrations of hypoxanthine and histidine, respectively, at early on-treatment. The cutoff value of (*p =* Responder) was 0.798. By using this model, the AUC was determined as 0.933 (95% CI, 0.734–0.996) for the validation set 1, with sensitivity of 92% and specificity of 88%, which indicates that the metabolite panel performed well in distinguishing responders and non-responders (Figure 3). A positive association between levels of the serum metabolite panel and PFS was highly statistically significant and independent (HR = 0.137, 95% CI, 0.040–0.467, *p =* 0.001) (Figure 4, Supplementary Table S6). Patients with high levels of hypoxanthine and histidine had a median PFS of 569 days (95% CI, 360–750 days), whereas the median PFS for patients with low levels of both metabolites was 126 days (95% CI, 42–256 days) (*p <* 0.001). Moreover, we found that high levels of the serum metabolite panel were significantly associated with improved overall survival of patient in the validation set 1 (*p =* 0.008) (Figure 6).

Another validation cohort was comprised of 10 patients with NSCLC treated with nivolumab (validation set 2) (Table 1). Serum samples were collected from these patients 2 weeks after the first infusion of nivolumab. Similar to the results of the discovery set and validation set 1, serum level of the two marker metabolites had a high sensitivity and specificity in discrimination of responders and non-responders of the validation set 2 (Figure 3). Responders had significantly higher levels of hypoxanthine and histidine in serum than non-responders (*p =* 0.019 and *p =* 0.038, respectively) (Figure 2), which were verified by the enzyme-based assays (Supplementary Figure S2) (Liu et al., 2009; Sun et al., 2012). We found that high levels of the serum metabolite panel were correlated with improved PFS in the validation set 2 (HR = 0.084, 95% CI, 0.009–0.762, *p =* 0.028) (Figure 4, Supplementary Table S6). Patients with high levels of the serum metabolite panel outlived patients with low levels of both metabolites, although the statistical significance cutoff was not met (*p =* 0.116) (Figure 6). Thus, the results from the two independent validation sets confirmed the serum metabolite panel as predictive biomarkers of NSCLC response to PD-1 blockade therapy.

## Discussion

Immunotherapies with checkpoint blockade antibodies targeting PD-1, PD-L1, and cytotoxic T-lymphocyte antigen 4 (CTLA-4) have remarkably improved the outcome of patients with advanced NSCLC and other cancers (Ribas and Wolchok, 2018). However, a substantial number of patients do not receive any clinical benefit, and robust predictors of therapeutic response are currently lacking. Though several tumor-derived and immune cell-derived biomarkers have been proposed, the demonstrated biomarker profiles often overlap between responders and non-responders and require invasive sampling from patients (Zhang et al., 2019). In the present study, we identified the serum metabolite biomarkers predictive of response to anti-PD-1 treatment in patients with advanced NSCLC based on metabolomic profiling using UHPLC-MS. The metabolomic profile of early on-treatment serum was found to be highly predictive of therapeutic responses, whereas the pretreatment serum metabolome was influenced by the initial states in patients including the treatments prior to PD-1 blockade, thereby making it less suitable for use. Indeed, we failed to identify reliable biomarkers from the pretreatment serum metabolomic data of the discovery cohort possibly due to the heterologous treatments prior to PD-1 blockade. Hypoxanthine and histidine in early on-treatment serum were identified and validated in independent patient cohorts as the predictive biomarkers of clinical outcomes of PD-1 block therapy. To our knowledge, this is the first report of the validated serum metabolite biomarkers predictive of response to immune checkpoint blockade therapies in lung cancer. This biomarker panel would enable the identification of patients who may benefit from continuing after the first administration of anti-PD-1 antibodies. Moreover, blood collection for these biomarkers is minimally invasive compared with the collection of tumor tissues. For clinical praxis, the nontargeted metabolomic approach can be replaced by a targeted MS analysis or sensitive enzyme-based assays specifically for the metabolite biomarkers, which could simplify the process and reduce the measurement costs and thus allow a more affordable, large-scale analysis (Cui et al., 2018).

The identified metabolite biomarkers, hypoxanthine and histidine, have values beyond their ability to predict the response to PD-1 blockade. They also offer novel insight into mechanisms of therapeutic resistance and suggest metabolic targets for interventions in combination with PD-1 blockade to improve clinical efficacy. Hypoxanthine is a key intermediate in adenosine metabolism, which can be synthesized from adenosine through sequential activities of adenosine deaminase (ADA) and purine nucleoside phosphorylase (PNP) (Boison and Yegutkin, 2019). We found that hypoxanthine levels were significantly higher in responders than in non-responders, which suggests a role of the adenosine-hypoxanthine metabolism in therapeutic resistance to PD-1 blockade. Previous studies have demonstrated that adenosine is implicated in the suppression of T cell-mediated antitumor responses, and the adenosine pathway has become an important therapeutic target in cancer (Li X et al., 2019). Several clinical trials have been initiated to test the efficacy of combined adenosine pathway inhibitors with PD-1/PD-L1 blockade in several cancers including NSCLC (Boison and Yegutkin, 2019). So far, much effort has been made to target adenosine-producing enzymes CD39 and CD73 and adenosine receptor A_2A_R for enhancing antitumor immunity (Arab and Hadjati, 2019). However, adenosine levels depend on the complex interplay between several adenosine-producing and -degrading pathways (Arab and Hadjati, 2019). Conversion of adenosine to hypoxanthine *via* ADA and PNP represents a currently underappreciated route that could regulate adenosine levels. Our finding prompts the intriguing question of whether increasing ADA and PNP activities for hypoxanthine synthesis from adenosine could improve antitumor immunity. In fact, ADA deficiency has been shown to result in tumor progression, and ADA activity of T cells has been suggested as an indicator of immune competence in patients with head and neck squamous cell carcinoma (Theodoraki et al., 2018).

Cancer cells increase uptake of amino acids, thereby depleting these resources for immune cells in the TME (Pavlova and Thompson, 2016). The amino acid transport and metabolism in T cells are also repressed by PD-1 ligation (Patsoukis et al., 2015). Thus, increased availability of amino acids may support the growth and function of T cells in the presence of PD-1 inhibitor. This may partially explain why responders had higher levers of serum histidine than non-responders among the patients receiving anti-PD-1 therapy. Moreover, high levels of histidine can increase the production of histamine through the reaction catalyzed by histidine decarboxylase (HDC). Histamine is an inhibitor of NADPH oxidase (NOX2) and has been approved in Europe in conjunction with interleukin-2 for relapse prevention in patients with acute myeloid leukemia (Stadtmauer, 2002). A recent study has shown that histamine targets myeloid-derived suppressor cells and improves the anti-tumor efficacy of PD-1/PD-L1 checkpoint blockade in mouse models (Grauers Wiktorin et al., 2019). Thus, our finding raises the possibility that supplementation of histamine/histidine and increasing HDC activity might be attractive strategies to enhance immunotherapy.

In conclusion, we report that the identification of metabolite biomarkers in early on-treatment serum constitutes a predictive tool for selecting NSCLC patients who stand to gain clinical benefit from anti-PD-1 therapy. Despite these provocative results, several limitations exist with this study. The patient cohorts in the current study were admittedly small and results need to be validated in large cohorts. Although the validation set one was comprised of patients receiving PD-1 inhibitor in combination with chemotherapy, the metabolite biomarkers need to be validated in more patient cohorts treated with combination therapy that is becoming a promising treatment strategy for NSCLC (Dong et al., 2019). Further studies are required to evaluate the serum metabolite panel for predicting clinical outcome of anti-PD-1 therapy in other cancer types. Our findings also warrant follow-up studies to check the ability of the serum metabolite biomarkers to predict the response to drugs targeting other immune-related proteins, such as PD-L1 and CTLA-4.

## Data Availability

The original contributions presented in the study are included in the article/Supplementary Material, further inquiries can be directed to the corresponding authors.
