# Supplementary material for: Serum Metabolite Biomarkers Predictive of Response to PD-1 Blockade Therapy in Non-Small Cell Lung Cancer
Source: Front Mol Biosci. 2021 May 21;8:678753. doi: 10.3389/fmolb.2021.678753 (PMC8176105; doi:10.3389/fmolb.2021.678753)
Supplement: Supplementary file 1 [file DataSheet1.PDF]

Supplementary Material

Supplementary Figure 1. Validation plot obtained from 200 times of permutation tests.

Supplementary Figure 2. Verification of higher levels of the serum marker metabolites in responders than in non-responders by the enzymatic assays.

Supplementary Table 1. Association between the therapeutic effect and clinical covariables in the discovery and validation sets.

Supplementary Table 2. Identified differential metabolites between responders and non-responders in the discovery set.

Supplementary Table 3. Binary logistic regression result based on the forward stepwise (Wald) method.

Supplementary Table 4. Univariable and multivariable analysis of PFS in the discovery set.

Supplementary Table 5. Univariable and multivariable analysis of OS in the discovery set.

Supplementary Table 6. Univariable and multivariable analysis of PFS in the validation sets.

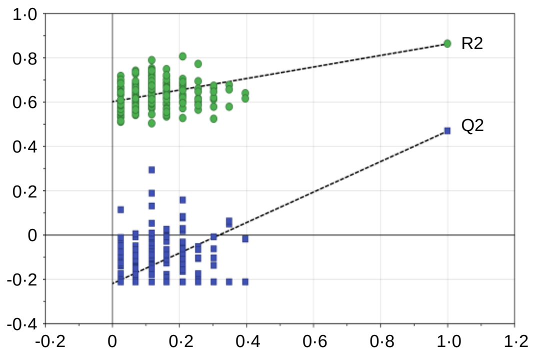

Supplementary Figure 1. Validation plot obtained from 200 times of permutation tests. The intercepts of R^2 = (0.0, 0.6) and Q^2 = (0.0, -0.21) illustrate that the OPLS-DA model is not over-fitting.

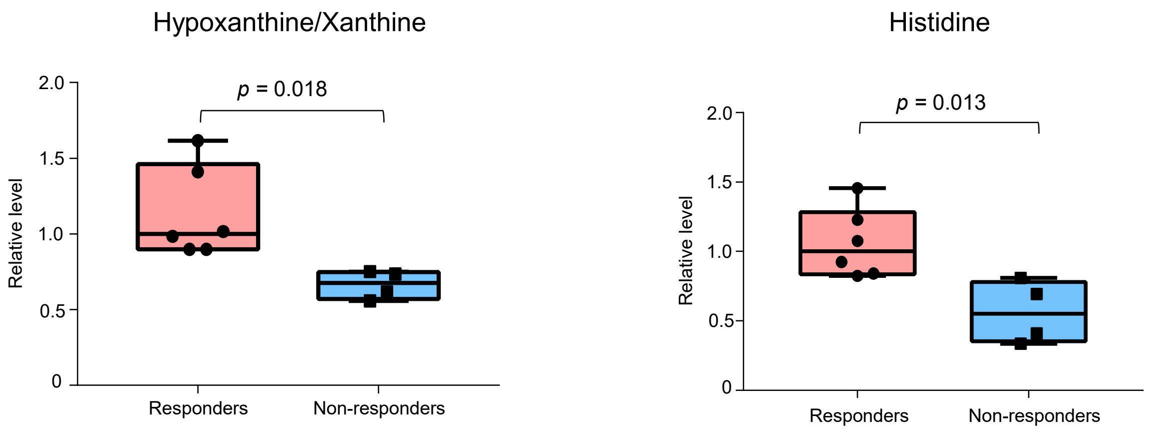

Supplementary Figure 2. Verification of higher levels of the serum marker metabolites in responders than in non-responders by the enzymatic assays. The serum samples from the validation set 2 were used for the enzymatic assays. Metabolite concentrations were normalized to the median value in the responder group. The box plots depict the minimum and maximum values (whiskers), the upper and lower quartiles, and the median. Groups were compared by Wilcoxon-Mann-Whitney test with Benjamini-Hochberg-based adjustment for multiple comparisons.

Supplementary Table 1. Association between the therapeutic effect and clinical covariables in the discovery and validation sets.

Characteristics	Discovery set (n = 43)			Validation set 1 (n = 21)			Validation set 2 (n = 10)		
	R (n = 21)	NR (n = 22)	p value[a]	R (n = 13)	NR (n = 8)	p value[a]	R (n = 4)	NR (n = 6)	p value[a]
Age									
≥65	5	8	0.370	5	1	0.336	1	4	0.524
< 65	16	14		8	7		3	2	
Sex									
Male	18	15	0.281	9	5	>0.999	4	5	>0.999
Female	3	7		4	3		0	1	
Smoking status									
Smoker	17	14	0.206	9	2	0.080	3	5	>0.999
Non-smoker	4	8		4	6		1	1	
Histology									
Squamous	8	9	0.850	13	8	>0.999	3	4	>0.999
Non-squamous	13	13		0	0		1	2	
Disease stage									
III	4	2	0.412	0	1	0.381	0	1	>0.999
IV	17	20		13	7		4	5	
Metastasis									
Yes	17	20	0.412	13	1	0.381	4	5	>0.999
No	4	2		0	7		0	1	
Radiotherapy									
Yes	9	6	0.231	1	3	0.266	4	4	0.467
No	11	16		6	3		0	2	
Unknown	1	0		6	2		0	0	
PD-L1 status[b]									
Negative	12	16	0.116	-	-	-	-	-	-
Positive	9	4		-	-		-	-	
Unknown	0	2		-	-		-	-	

[a] P value represents Fisher's exact test or Chi-square function whenever required.

[b] Immunohistochemistry cutoff for positivity on tumor cells of 1% or higher.

Supplementary Table 2. Identified differential metabolites between responders and non-responders in the discovery set.

**Metabolites**	**ESI mode**	**VIP**	***p* value**	**FDR**	**FC[a]**	**HMDB**	**Compound category[b]**
Cystine	−	1.114	0.008	0.016	-0.356	HMDB0000192	Amino acids
Threonine	+	1.602	<0.001	<0.001	-0.468	HMDB0000167	Amino acids
Histidine	+	1.513	<0.001	<0.001	-0.565	HMDB0000177	Amino acids
3-Oxotetradecanoic acid	−	1.017	0.024	0.029	0.756	HMDB0010730	Fatty acids and conjugates
1,7-Dimethyluric acid	−	1.168	0.016	0.017	-1.370	HMDB0011103	Nucleotide and derivatives
Hypoxanthine	+	1.061	<0.001	<0.001	-0.581	HMDB0000157	Nucleotide and derivatives

[a] the ratio of mean values of metabolite levels in non-responders to those of responders, and then log2 transformed.

[b] the chemical taxonomy in HMDB database.

FC, fold change.

Supplementary Table 3. Binary logistic regression result based on the forward stepwise (Wald) method.

	**B**	**S.E.**	**Wald**	***p* value**
Hypoxanthine	2.159	0.811	7.088	0.008
Histidine	2.159	0.811	7.088	0.008
Constant	-2.176	0.726	8.989	0.003

B, Binary logistic regression coefficient; S.E., Standard errors.

Supplementary Table 4. Univariable and multivariable analysis of PFS in the discovery set.

Factors	Univariable analysis		Multivariable analysis	
	HR (95% CI)	*p* value	HR (95% CI)	*p* value
Age, ≥ 65 vs. < 65	0.833 (0.387-1.790)	0.639		
Sex, Female vs. male	0.655 (0.304-1.409)	0.279		
Histology,				
Non-squamous vs.Squamous	1.054 (0.527-2.108)	0.882		
Smoke,				
Smoker vs. Non-smoker	0.600 (0.291-1.238)	0.167		
Disease stage, IV vs. III	4.049 (0.962-17.035)	0.056		
Metastasis, Yes vs. No	4.049 (0.962-17.035)	0.056		
Radiotherapy, Yes vs. No	1.041 (0.511-2.122)	0.912		
PD-L1 status, Positive vs. Negative	0.431 (0.191-0.974)	0.043	0.653 (0.264-1.619)	0.358
Hypoxanthine, High vs. Low	0.215 (0.102-0.449)	< 0.001		
Histidine, High vs. Low	0.336 (0.166-0.679)	0.002		
Combination,				
High in 1 vs. Low in 2	0.173 (0.069-0.430)	< 0.001	0.170 (0.067-0.431)	< 0.001
High in 2 vs. Low in 2	0.078 (0.027-0.221)	< 0.001	0.094 (0.031-0.289)	< 0.001

Supplementary Table 5. Univariable and multivariable analysis of OS in the discovery set.

Factors	Univariable analysis		Multivariable analysis	
	HR (95% CI)	*p* value	HR (95% CI)	*p* value
Age, ≥ 65 vs. < 65	1.194 (0.467-3.057)	0.711		
Sex, Female vs. male	1.036 (0.379-2.827)	0.945		
Histology,				
Non-squamous vs.Squamous	0.947 (0.396-2.267)	0.903		
Smoke,				
Smoker vs. Non-smoker	0.916 (0.357-2.354)	0.856		
Disease stage, IV vs. III	1.745 (0.406-7.498)	0.454		
Metastasis, Yes vs. No	1.745 (0.406-7.498)	0.454		
Radiotherapy, Yes vs. No	1.286 (0.529-3.127)	0.579		
PD-L1 status, Positive vs. Negative	0.285 (0.082-0.986)	0.047	0.494 (0.123-1.985)	0.320
Hypoxanthine, High vs. Low	0.247 (0.099-0.449)	0.003		
Histidine, High vs. Low	0.298 (0.121-0.737)	0.009		
Combination,				
High in 1 vs. Low in 2	0.209 (0.076-0.578)	0.003	0.249 (0.087-0.707)	0.009
High in 2 vs. Low in 2	0.124 (0.039-0.397)	< 0.001	0.194 (0.051-0.730)	0.015

Supplementary Table 6. Univariable and multivariable analysis of PFS in the validation sets.

Factors	Validation set 1				Validation set 2			
	Univariable analysis		Multivariable analysis		Univariable analysis		Multivariable analysis	
	HR (95% CI)	*p* value	HR (95% CI)	*p* value	HR (95%CI)	*p* value	HR (95% CI)	*p* value
Age, ≥ 65 vs. < 65	1.437 (0.467-4.419)	0.527			0.919 (0.239-3.536)	0.902		
Sex, Female vs. male	0.583 (0.195-1.744)	0.335			0.111 (0.007-1.776)	0.120		
Histology,								
Non-squamous vs.Squamous	-	-			1.43 (0.289-7.082)	0.661		
Smoke,								
Smoker vs. Non-smoker	0.261 (0.079-0.862)	0.028	0.532 (0.139-2.039)	0.357	2.599 (0.310-21.799)	0.379		
Disease stage, IV vs. III	-	-			0.579 (0.064-5.230)	0.627		
Metastasis, Yes vs. No	-	-			0.579 (0.064-5.230)	0.627		
Radiotherapy, Yes vs. No	-	-			0.297 (0.049-1.797)	0.186		
Hypoxanthine and Histidine,								
High vs. Low	0.137 (0.040-0.467)	0.001	0.184 (0.047-0.715)	0.014	0.084 (0.009-0.762)	0.028		
